# Lipoprotein metabolism in nonalcoholic fatty liver disease

**DOI:** 10.7555/JBR.27.20120077

**Published:** 2012-12-01

**Authors:** Zhenghui Gordon Jiang, Simon C. Robson, Zemin Yao

**Affiliations:** a Department of Medicine, Beth Israel Deaconess Medical Center/Harvard Medical School, Boston, MA, USA;; b Division of Gastroenterology, CLS612, Beth Israel Deaconess Medical Center/Harvard Medical School, Boston, MA, USA;; c Department of Biochemistry, Microbiology and Immunology, Ottawa Institute of Systems Biology, University of Ottawa, Ottawa, Ontario KIH 8M5, Canada.

**Keywords:** nonalcoholic fatty liver disease (NAFLD), hepatic steatosis, nonalcoholic steatohepatitis, apolipoprotein, lipoprotein metabolism, very low density lipoprotein

## Abstract

Nonalcoholic fatty liver disease (NAFLD), an escalating health problem worldwide, covers a spectrum of pathologies characterized by fatty accumulation in hepatocytes in early stages, with potential progression to liver inflammation, fibrosis, and failure. A close, yet poorly understood link exists between NAFLD and dyslipidemia, a constellation of abnormalities in plasma lipoproteins including triglyceride-rich very low density lipoproteins. Apolipoproteins are a group of primarily liver-derived proteins found in serum lipoproteins; they not only play an extracellular role in lipid transport between vital organs through circulation, but also play an important intracellular role in hepatic lipoprotein assembly and secretion. The liver functions as the central hub for lipoprotein metabolism, as it dictates lipoprotein production and to a significant extent modulates lipoprotein clearance. Lipoprotein metabolism is an integral component of hepatocellular lipid homeostasis and is implicated in the pathogenesis, potential diagnosis, and treatment of NAFLD.

## INTRODUCTION

An important evolutionary feature of vertebrates is the emergence of the liver, a versatile organ that fulfills numerous physiological needs and, especially, the maintenance of metabolic homeostasis. The regulation of lipid metabolism is one of the liver's core functions. The liver can both acquire lipids from the circulation and secrete lipid particles into the blood stream. Some species, such as the shark, have a hypertrophic liver reaching 10%–20% of total body weight; thus up to half of the liver mass is composed of fat and provides buoyancy functions[1]. Migratory birds reversibly accumulate lipids in the liver, as well as the peripheral adipose tissue in the autumn, to meet the extreme metabolic demand in the winter, a phenomenon exploited by humans to produce the *foie gras*. On the other hand, the human liver does not seem to be designed for much lipid storage. Excessive fatty accumulation in the liver is recognized as a pathological state. Fatty liver may occur acutely in pregnant women with genetic deficiency of 3-hydroxyacyl CoA dehydrogenase, an enzyme involved in β-oxidation of fatty acids, resulting in accumulation of medium to long chain fatty acids and subsequent acute liver failure[2].

The pathology of chronic fatty accumulation was first characterized in alcoholism, a chronic condition associated with the potential of acute hepatitis exacerbation[3]. It was first described by Jurgen Ludwig in 1981 that the identical fatty accumulation pathology also occurs in nonalcoholics[4]. Nonalcoholic fatty liver disease (NAFLD) is a condition characterized by histological findings of fatty accumulation in hepatocytes that are indistinguishable from alcoholic fatty liver disease. NAFLD covers a broad spectrum of disease states, ranging from fatty infiltration (steatosis), inflammation [steatohepatitis, also known as nonalcoholic steatohepatitis (NASH)], to fibrosis and cirrhosis[5]. NAFLD is now recognized as an escalating health problem, affecting both affluent as well as developing countries[6],[7]. Recent epidemiological studies indicate that the prevalence of NAFLD in the United States is 33% determined by ^1^H NMR spectroscopy[8], and 46% determined by ultrasound[9]. The prevalence among Hispanics and Caucasian males was found to be the highest[10]. NAFLD is a hallmark of metabolic syndrome, with approximately 70% obese and diabetic patients also having NAFLD[11]–[13]. Contrary to an early notion that fatty liver was a benign condition, about 30% of patients with positive ultrasound findings of fatty liver showed NASH on biopsy[9]. In another study, 3% of NAFLD patients develop cirrhosis, a rate much higher than the general public[[14]]. This translates into 10%–14% of the general population having NASH and 1%–1.4% having NASH induced cirrhosis. Other studies showed that hepatic steatosis is an independent risk factor for coronary artery disease, the leading cause of mortality in developed countries[15]–[17]. In liver transplant, hepatic steatosis is a well-established risk factor for ischemia reperfusion injury, leading to poor outcome of the graft[18].

Patients with NAFLD are frequently asymptomatic. Physical findings are rare before impaired compensation of the liver functions. Clinically, the workup of NAFLD is often triggered by elevated liver enzymes after ruling out alcohol abuse or viral hepatitis. Aspartate aminotransferase to alanine aminotransferase ratio is usually less than one until advanced fibrosis has occurred[19]]. Hepatic steatosis is also a frequent incidental finding during abdominal imaging studies. Ultrasonography is safe and cost-effective, thus is often used as a screening tool. On ultrasonography, the steatosis produces a diffuse increase in echogenicity compared to the neighboring renal cortex as a result of increased parenchymal reflectivity of intracellular fat inclusions[20]. A recent report showed that ultrasound has a sensitivity of 92% and specificity of 100% in detecting hepatic steatosis after workup for other causes of liver diseases[21]. Computed tomography and magnetic resonance imaging also offer information on hepatic steatosis. Proton magnetic resonance spectroscopy (^1^H MRS) has been gaining popularity in epidemiological research, as it offers the most sensitive and precise quantification of steatosis[22],[23]. Despite advances in imaging modalities, liver biopsy is still the gold standard in the diagnosis of NAFLD as it is the only way to make the distinction between simple steatosis versus steatohepatitis or cirrhosis. Besides the invasive nature of biopsy, sampling and interpretation variability limits the accuracy of NALFD staging[24].

This review summarizes our current understanding of the relationship between lipoprotein metabolism and NAFLD, with a goal of clarifying pathophysiology of the disease and potentially identifying new diagnostic strategies and therapeutic targets.

## LIPOPROTEIN METABOLISM AND PATHOPHYSIOLOGY OF NAFLD

**Fig. 1:**
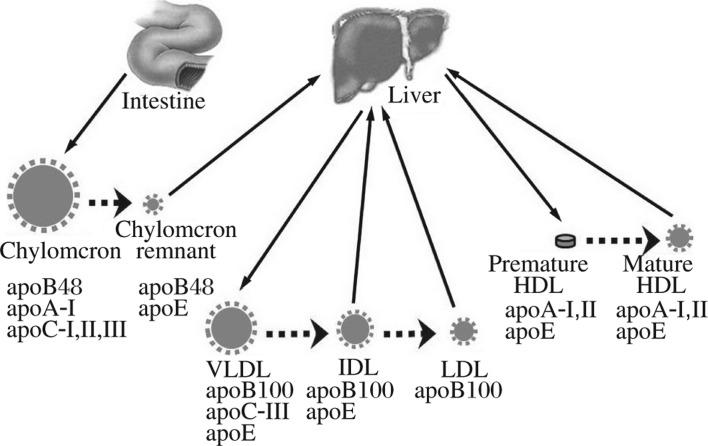
Overview of lipoprotein metabolism. Human lipoproteins are predominantly produced by the small intestine and the liver. Small intestine produces chylomicron, which contains apoB48, apoA-I, apoC-I, apoC-II, and apoC-III. The remnant particles, after utilization of lipids by the peripheral tissue, are taken up by the hepatocytes. The liver produces apoB-100-containing VLDL and premature HDL. VLDL is hydrolyzed in circulation and converted into IDL and LDL. Both IDL and LDL can be taken up by the hepatocytes. The discoidal shaped premature HDL becomes mature HDL in the circulation, and serves an important role in reverse cholesterol transport. VLDL: very low density lipoprotein; HDL: high density lipoproteins; IDL: intermediate density lipoprotein; LDL: low density lipoprotein.

Hydrophobic lipids are transported in the form of lipoproteins. The vehicles of this rather complex transport system consist of apolipoproteins, a family of surface active proteins synthesized predominantly by the liver and, to a lesser extent, by the small intestine. The three major classes of lipoproteins in humans are chylomicrons, very low density lipoprotein (VLDL), and high density lipoprotein (HDL) (***Fig. 1***). Chylomicrons are synthesized in the small intestine, whereas VLDL is produced by the liver and is the precursor of low density lipoprotein (LDL). Both chylomicron and VLDL particles contain a single copy of apolipoprotein B (apoB), an extremely hydrophobic and tightly-bound protein that is often referred to as a nonexchangeable apolipoprotein. The major exchangeable apolipoproteins include apoA-I, apoA-II, apoA-IV, apoA-V, apoC-I, apoC-II, apoC-III, and apoE[25],[26]. These are smaller proteins (as compared to apoB) and are often co-secreted with apoB-containing lipoproteins such as VLDL and chylomicrons. Some of these proteins, such as apoA-I, are also produced as lipid-poor forms and constituents of the pre-β-HDL, which later matures into HDL during the reverse cholesterol transport process. The only route for triglyceride (TAG) export from hepatocytes is via the assembly of VLDL (see below). Serum lipoprotein is also a major source of hepatocellular lipid uptake, including chylomicron remnant, LDL and HDL.

The cause of NAFLD remains poorly understood. Nonetheless, it can be rationalized that hepatic steatosis is a result of net hepatocellular retention of lipids, especially in the form of TAG. From a liver centric point of view, this imbalance results from abnormalities in one or more of the following four processes: (1) hepatic uptake of fatty acid, lipoprotein and glucose, (2) *de novo* TAG synthesis, (3) TAG degradation and fatty acid β oxidation, and (4) lipoprotein secretion in the form of VLDL[27]. In NAFLD patients, 15% of the hepatic lipid comes from dietary fatty acid, 30% from *de novo* synthesis, and 60% from the lipolysis of adipose tissue[28].

The most reproducible observation of NAFLD is its association with metabolic syndrome and insulin resistance. Accumulating evidence has revealed that insulin resistance profoundly affects hepatic lipid homeostasis[29]. Under insulin resistance state, all of the three major sources of hepatic TAG increased, namely, the albumin bound free fatty acid (FFA) from lipolysis of adipose tissue, the fatty acids metabolized from circulating chylomicrons and VLDL-derived lipoproteins via lipoprotein lipase (LPL), and fatty acids from de novo lipogenesis[29]. Insulin modulates lipolysis of adipose tissue, leading to increased levels of serum FFAs[30]. Insulin resistance also increases the production of chylomicron and VLDL, but hampers their lipolysis in the circulation, thus increasing the hepatic uptake of lipids from chylomicron and VLDL[31],[32]. Persistent elevation of serum LDL leads to the accumulation of plasma oxidized LDL (oxLDL), which is one of the endogenous antigens in a number of diseases featured by oxidative stress. A high titer of serum IgA to oxLDL level was associated with worsening fatty liver disease[33]. Circulating IgG against lipid peroxidation products was higher in NAFLD patients than in controls in an independent study[34]. In particular, an elevated anti-malondialdehyde antibody level was correlated with an increased risk of cirrhosis[34].

Closely related with insulin resistance is adipocyte dysfunction, an important metabolic derangement leading to hepatic steatosis. Leptin is an adipocyte-derived hormone with pleiotropic physiological functions, most importantly known to exert an effect on the hypothalamus[35]. The leptin deficient mouse (ob/ob) develops massive fatty liver as well as obesity and insulin resistance[36]. In a transgenic mouse model, symptoms resembling congenital generalized lipodystrophy were observed by expressing constitutively activated sterol regulatory element-binding protein 1c (SREBP-1c)[37]. The lipodystrophic mice, despite normal weight, had phenotypes similar to metabolic syndrome, including massive fatty liver. Infusion of leptin is able to reverse the phenotype of hepatic steatosis and insulin resistance. Similar effects of leptin on alleviating hepatic steatosis have also been illustrated in lipodystrophic patients[38],[39]. However, cellular and molecular mechanisms by which leptin exerts its effect on regulating hepatic lipoprotein production remain to be defined.

## APOLIPOPROTEIN B

**Fig. 2:**
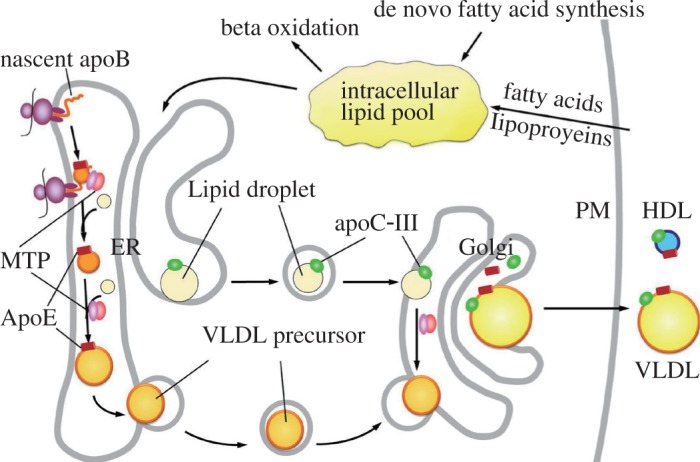
VLDL synthesis in hepatocytes. VLDL synthesis requires a synchronized process along apoB-100 maturation in the ER-Golgi secretory compartments. The nascent apoB-100 forms a primordial particle within the ER with the assistance of MTP. The primordial particle subsequently expands as the translation of apoB continues and more lipids are added. Intralumenal lipid droplets are also formed within the ER, and carry apoC-III. The VLDL precursor will combine with the intralumenal lipid droplets, either in the ER or in the Golgi, through a process that may or may not require the MTP activity. Mature VLDLs are secreted through vesicle-mediated exocytosis. PM: plasma membrane; VLDL: very low density lipoprotein; ER: endoplasmic reticulum; MTP: microsomal triglyceride-transfer protein.

The mature apoB-100 consists of 4,536 amino acids and is one of the largest proteins secreted by the liver in the form of VLDL[40],[41]. The small intestine secrets chylomicrons that contain apoB-48, which represents the *N*-terminal 48% of apoB-100 (resulting from the *apoB* mRNA editing)[42],[43]. The assembly of apoB-48 and apoB-100 with lipids dictates the production of respective chylomicrons and VLDL, a process involving a complex folding and intracellular trafficking pathway and requires coordination of multiple cellular machineries (***Fig. 2***)[44]. Genetic defects in the APOB gens can lead to NAFLD, as exemplified in familial hypobetalipoproteinemia (FHBL)[45],[46]. FHBL is an autosomal co-dominant disease, characterized by less than 5^th^ percentile of total cholesterol, LDL cholesterol, or total apoB[47]. The estimated prevalence of heterozygotic forms of FHBL, based on clinical criteria, is 1/500-1/1,000[48]. The homozygotic form of FHBL is exceedingly rare and often causes significant developmental defects. This underlying genetic condition either directly or indirectly affects the integrity of the apoB secretory pathway. A number of truncating or missense mutations within the APOB gene were found to cause FHBL[49]. Interestingly, longevity is reportedly associated with FHBL, probably because the lowered serum cholesterol in FHBL protects against cardiovascular diseases[50].

Although many FHBL individuals are clinically silent, FHBL significantly increases the susceptibility for NAFLD. This has been shown in mice expressing truncated forms of apoB[51],[52]. In humans, FHBL is found to cause an increased hepatic TAG content and elevation in liver enzymes[53]–[55]. It is unclear whether hepatic steatosis in FHBL individuals follows the same natural history as those NAFLD patients without FHBL. It has been shown, however, that FHBL-induced hepatic steatosis in individuals does not necessarily cause insulin resistance[56]. A non-obese FHBL patient with cirrhosis developed severe NASH after receiving liver transplant from a healthy FHBL donor[57], suggesting that NAFLD can progress aggressively in FHBL without insulin resistance. However, whether or not steatosis alone (without insulin resistance) is sufficient to drive hepatic inflammation remains to be determined. It should be pointed out that not all FHBL mutations cause hepatic steatosis. In a phase III clinical trial using apoB antisense oligonucleotide, patients with familial hypercholesterolemia receiving the inhibitor did not develop hepatic steatosis after 13 weeks of treatment[58]. There is likely a spectrum of severities in steatosis, depending upon the degree of impairment in the secretion of apoB-containing lipoproteins.

**Fig. 3:**
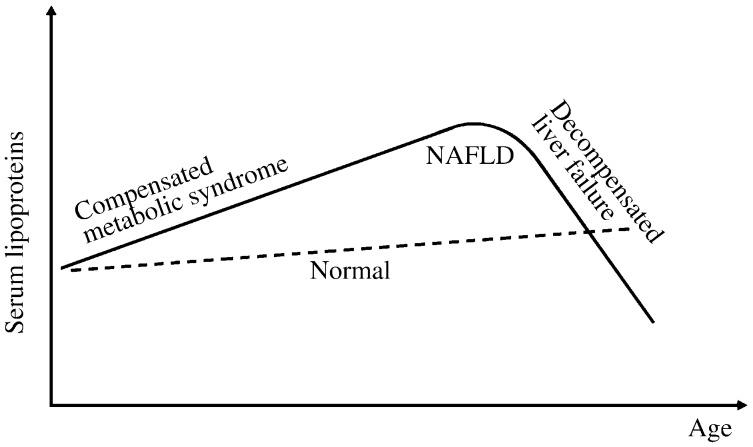
Predicted natural history of serum lipoprotein levels in NAFLD. NAFLD is associated with dyslipidemia, an integral feature of metabolic syndrome. As disease progresses, hepatic lipoprotein production will decline as a result of decompensated liver failure. This progression may be manifested as a decline in serum lipoprotein levels, which may potentially precede global decline in hepatic synthetic function, a condition currently monitored by albumin and coagulation time clinically. NAFLD: non-alcoholic fatty liver disease.

Currently, it is unclear whether or not development of NAFLD alone can cause impairment in VLDL secretion. Insulin resistance, generally, is associated with increased hepatic TAG production in the form of VLDL, as dyslipidemia is a hallmark of metabolic syndrome[59],[60]. Increased hepatic fat content often (but not always) correlates with increased hepatic TAG secretion[60],[61]. However, as NAFLD progresses, it is conceivable that the production of VLDL/apoB might decrease secondary to the impairment of hepatocellular function (***Fig. 3***). Studies of seven patients with biopsy proven NASH showed decreased apoB production rate (by 50%) as compared with obese or lean controls without NAFLD[62]. One theory for this impairment is endoplasmic reticulum (ER) stress. It has been shown in cell culture and animal studies that, although moderate FFA overload increases apoB secretion, prolonged exposure of FFA induced ER stress and resulted in decreased apoB secretion[63]. The ER stress may represent a state prior to the decline of global hepatic synthetic function, a condition of end-stage liver disease characterized by extensive replacement of hepatocytes by fibrotic tissues (which is clinically being monitored by albumin or coagulation factors) (***Fig. 3***). Effective apoB maturation in conjunction with VLDL assembly and secretion, however, demands a higher level of cellular function integrity, a process far more complex than the secretion of soluble proteins such as albumin or coagulation factors. If the decline of VLDL production does precede the impairment in global hepatic synthetic function, one might expect that apoB production can serve as a potential surrogate marker to replace the rather invasive liver biopsy for monitoring NAFLD disease progression. However, the challenge in measuring apoB production is the labor intensive nature of apoB kinetic studies. The gold standard to measure apoB production rate requires the use of isotopic labeled amino acids or lipids, a rather cumbersome method. Validated surrogate markers could potentially replace these methods.

## MICROSOMAL TRIGLYCERIDE TRA-NSFER PROTEIN

Although not an apolipoprotein, the microsomal triglyceride-transfer protein (MTP) plays an indispensable role in apoB metabolism[64]. Synthesized mainly in the liver and small intestine, MTP exists as a heterodimer through non-covalent binding to protein disulfide isomerase, an ER resident protein[65]. The amino acid sequence of MTP is homologous to the *N*-terminal 20% of apoB, suggesting that MTP and apoB may share a common evolution origin[66],[67]. MTP was found capable of transferring TAG between vesicles in vitro, and was thus proposed as an lipid transfer protein during apoB maturation[68]. MTP binds to apoB and ER membrane, and also functions as a chaperone that assists in apoB folding (***Fig. 2***)[69],[70]. The lack of functional MTP causes abetalipoproteinemia, a condition characterized by the virtual absence of apoB-containing lipoprotein in the plasma[71]. The hallmark clinical manifestations of abetalipoproteinemia include hepatic steatosis as well as developmental defects associated with the malabsorption of lipid-soluble vitamins[72],[73].

Abetalipoproteinemia is a rare, naturally occurring cause of NAFLD. The MTP inhibition can also be acquired in viral hepatitis or iatrogenic by MTP inhibitors. Hepatitis C virus genotype 3 (HCV-3) is known to cause hepatic steatosis. In HCV-3 induced steatosis, the MTP activity and the *MTTP* mRNA level were both reduced[74]. There was a positive correlation between the histological grade of steatosis and reduction in the *MTTP* mRNA[74]. The MTP inhibitors were developed initially with a hope to treat hypercholesterolemia. However, although the MTP inhibitors effectively lower serum LDL, they cause a dose-dependent hepatic steatosis and variable severity of transaminitis[75]. These side effects have prohibited the FDA approval of first generation MTP inhibitors for the treatment of dyslipidemia. However, the efforts to optimize and balance the efficacy and adverse profile of MTP inhibitors have not ceased[76].

Genetic studies have identified an interaction between MTP and NAFLD. A polymorphism at the promoter region (-493G/T) of MTTP is associated with biological surrogates of steatohepatitis in patients with type II diabetes[77]. The G allele is responsible for decreased MTP transcription, and is prone to increased intrahepatic TAG content. The TT polymorphism is associated with an atherogenic postprandial lipid profile, elevated levels of serum hs-CRP and resistin, and an increased risk of coronary artery diseases[78],[79]. This is an example of genetic differences accounting for risk factors of divergent metabolic disorders including fatty liver and coronary artery disease. MTP also plays a role linking insulin resistance to hepatic VLDL production. The *MTTP* gene expression is under negative regulation of insulin[80]. Insulin leads to the phosphorylation of forkhead transcription factor FoxO1 and its exclusion from the nucleus, resulting in the inhibition of MTP transcription[81]. Insulin also inhibits MTP production via the MAPK pathway[82]. It could therefore be postulated that one effect of insulin resistance is the loss of such negative regulation, leading to increased MTP production, thus more pronounced apoB-VLDL production.

## APOLIPOPROTEIN C-III

There is a recent revival of interests in apoC-III, an exchangeable apolipoprotein, for its newly suggested role in lipoprotein metabolism and NAFLD. Human apoC-III is a 79-amino acid glycoprotein found in all three classes of lipoproteins, VLDL, chylomicron and HDL[26],[83]. The liver contributes to the majority of serum apoC-III in the form of VLDL. Like other exchangeable apolipoproteins, apoC-III can be transferred between lipoprotein particles. The NMR structure of human apoC-III on lipid-mimicking SDS micelles showed an extended helical belt structure, wrapping over the surface of the micelle as an open necklace[84]. Similar extended alpha helical conformation is also seen in recently determined apoA-I and apoA-IV structures[85],[86].

**Fig. 4:**
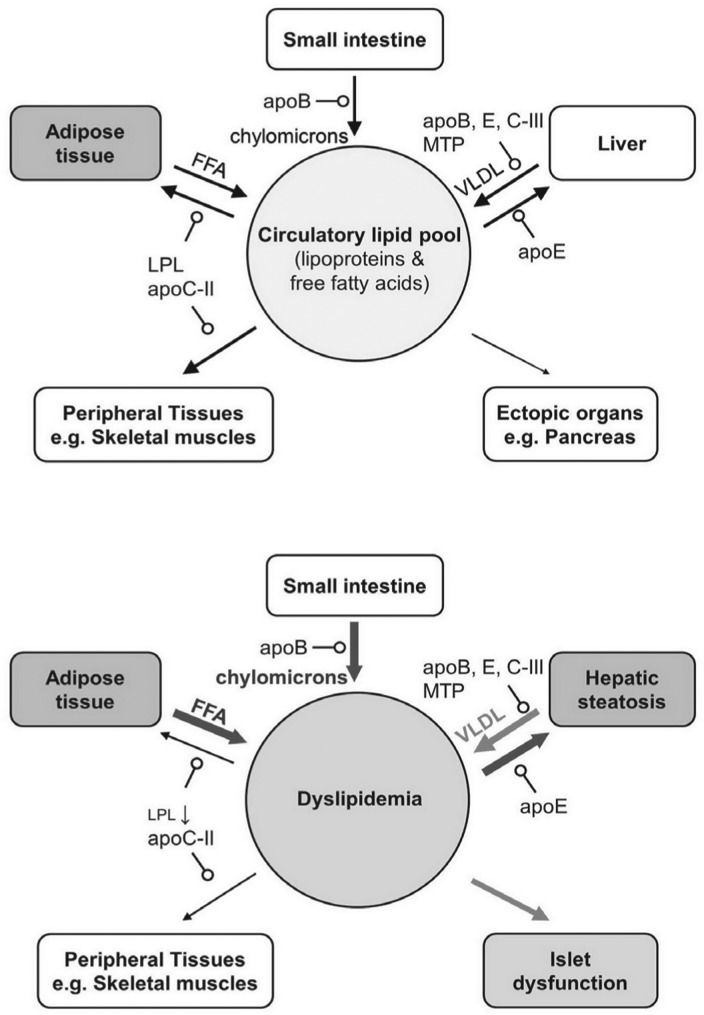
Lipid homeostasis in normal state (A) vs insulin resistance state (B). The flow of lipids among different organs is shown schematically. The pointed-arrow depicts the direction of lipid flow; the line thickness and grayscale represent the relative quantity. The arrow with open circle indicates factors mediating the lipid delivery. In normal lipid homeostasis, dietary lipids are excreted with apoB-48 in the forms of chylomicrons from the small intestine. The majority of this lipid load is utilized by peripheral tissues (e.g. adipocytes and skeletal muscles). A portion of this lipid load reaches the liver via apoE mediated uptake. Under insulin resistance conditions, besides increased FFA release from adipocytes, decreased LPL activity shunts more lipid load away from the peripheral tissues toward ectopic organs such as the liver and the pancreas. Hepatic steatosis is a high lipid turnover state, a condition associated with increased uptake and secretion of lipoproteins. Fatty deposition in islet cells further exacerbates metabolic syndrome. VLDL: very low density lipoprotein; FFA: free fatty acid; LPL: lipoprotein lipase.

The plasma concentration of apoC-III is closely associated with plasma concentrations of TAG. It was initially proposed that apoC-III inhibits LPL-mediated catabolism of TAG-rich lipoproteins, thus resulting in increased plasma TAG concentration[87]. Recently, it was found that apoC-III acts intracellularly and stimulates hepatic secretion of TAG-rich VLDL under lipid-rich conditions (***Fig. 2*** and ***Fig. 4***)[88]. This effect was seen in cell cultures and *apoc3*-knockout mice infected with adenoviruses expressing human apoC-III[88]–[90]. Metabolic labeling studies showed that apoC-III stimulates the incorporation of newly synthesized TAG into the microsomal compartments[88]. ApoC-III may also facilitate the expansion of nascent VLDL particles, as such function is abolished by a naturally occurring A23T mutation linked to hypotriglyceridemia in humans[90]. Furthermore, apoC-III appears to link lipoprotein metabolism to glucose metabolism. Glucose induces apoC-III transcription, while PPARα, PPARγ, Rev-Erb, FXR, and insulin all exert an inhibitory role in apoC-III transcription[91]. Therefore, apoC-III could underlie the adaptive effect of TAG hyper-secretion in the state of metabolic syndrome.

It remains controversial as to whether genetic variants of apoC-III are directly implicated in NAFLD. Over-expression of human apoC-III in transgenic mice predisposes the animal to diet induced hepatic steatosis and hepatic insulin resistance[92]. Genetic studies in humans showed that multiple polymorphisms in the promoter region of APOC3 were associated with familial hypercholesterolemia[93]–[95]. Among these polymorphisms, T-455C and C-482T were located in the insulin response element, a region that exerts negative regulation on apoC-III expression[93],[96]. It was reported that T-455C and C-482T were associated with hepatic steatosis in 95 healthy Asian Indian men, as well as a test group composed of 163 healthy non-Asian Indian men[23]. However, the association between the APOC3 promoter polymorphism and NAFLD was not observed in the Dallas Heart Study, in which a cohort of more African Americans with older age and higher BMI were studied[22]. The difference in population characteristics may explain discrepancies between the two studies. Nevertheless, the debate on SNP association does not negate the role of apoC-III in TAG-rich VLDL metabolism, which likely has an impact on hepatic steatosis.

## APOLIPOPROTEIN E

Apolipoprotein E (apoE) is a 299-amino acid protein, initially discovered as an exchangeable apolipoprotein that modulates lipoprotein metabolism[97] and also plays an important role in neurodegenerative diseases such as Alzheimer's disease[98]. Serum apoE is produced primarily by hepatocytes either in the form of VLDL or in association with HDLs. In addition to the liver, apoE is also expressed in a variety of other tissues including the brain, spleen, lung, adrenal gland, ovary kidney and macrophages[97]. There are three isoforms of human apoE, ϵ2, ϵ3, and ϵ4, which differ in amino acids at positions 112 and 158 (ϵ2, Cys and Cys; ϵ3, Cys and Arg; ϵ4, Arg and Arg)[99]. The ϵ2 genotype is linked to type III hyperlipidemia, a familial condition associated with premature atheroscloerosis[100],[101].

ApoE is implicated in lipoprotein metabolism both extracellularly and intracellularly. ApoE is present on chylomicrons, but its mRNA is not found in enterocytes. ApoE becomes chylomicron-bound in the circulation and mediates the removal chylomicron remnant via the LDL receptor (LDLR) (***Fig. 4***)[102],[103]. The *N*-terminal half of the apoE protein shares high identity to the LDLR binding domain in apoB, thus conferring its LDLR binding ability[104]. The affinity of apoE to LDLR is 25-fold higher than that of apoB[105]. ApoE also interacts with receptors other than LDLR, such as LDL receptor related protein 1 (LRP1), apoE receptor 2 (apoER2) and the VLDL receptor[98]. Thus, the absence of LDLR does not entirely affect chylomicron clearance[106].

Intracellularly, apoE facilitates apoB maturation and VLDL assembly in hepatocytes. Expression of apoE in immortalized human or rat hepatocytes stimulates VLDL secretion[107],[108]. The apoe-null mouse showed markedly reduced VLDL production from the liver[109], which could be restored by adenovirus-mediated apoE3 expression[108]. Although the exact mechanism of this stimulatory effect is unclear, current evidence suggests that apoE impacts the early assembly of VLDL in the ER rather than the VLDL maturation during post-ER trafficking (***Fig. 2***)[110],[111].

The overall impact of apoE in NAFLD reflects its dual roles in lipoprotein metabolism, the secretion of VLDL and clearance of postprandial chylomicrons. The hepatic TAG content was markedly increased in the apoe-null mouse, with a perivenous distribution of steatosis on biopsy[111]. Nevertheless, the apoe-null mouse is more resistant to diet-induced NAFLD than the wildtype mouse, likely as a result of impaired postprandial uptake of chylomicrons by the hepatocytes[112]. The extent of diet induced steatosis in apoe-null mouse is less than ldlr-null mouse, suggesting the presence of an apoE-mediated hepatic TAG uptake pathway independent from LDLR.

In humans, the impact of apoE on hepatic steatosis varies among different apoE isoforms. In a case control study of 57 biopsy confirmed NASH patients, the *APOE ϵ3* allele was overrepresented in NASH patients[113]. The ϵ3/ϵ3 genotype was strongly associated with increased risk of NASH (odds ratio = 7.941; p = 0.000)[113]. In other case-control studies, the ϵ2 and ϵ4 allele carriers had significant reduction in NAFLD risk[114],[115]. The hepatic uptake of lipids from dietary sources is in competition with non-hepatic utilization via the lipoprotein lipase (***Fig. 4***). It could be postulated that in metabolic syndrome, a condition associated with dietary fat overload, apoE may act as a mediator that regulates the distribution of dietary lipid load between the liver and the peripheral tissue. If this hypothesis is true, there might be a role for therapeutic strategies targeting the apoE-receptor interaction, thereby modulating this balance.

## OTHER APOLIPOPROTEINS, LIPOPROTEIN LIPASES AND NUCLEAR HORMONE RECEPTORS

The roles that other exchangeable apolipoproteins play in NAFLD are less well defined. Proteomics analysis of serum samples from 65 NAFLD patients (with varying biopsy proven stages of disease) showed that several apolipoproteins have >14% differences (*P* < 0.05) in their serum concentration as compared with 16 obese controls, including apoA-II, apoA-IV, apoB, apoC-I, apoC-III and apoL-I[116]. However, several apolipoproteins (such as apoA-I and apoA-V) and lipases (such as LPL) that are potentially involved in NAFLD were not identified in this study.

Apolipoprotein A-I is the most important apolipoprotein in HDL, and plays essential roles in reverse cholesterol transport as well as a number of physiological functions that are considered cardioprotective[117]. Low HDL and high apoB/apoA-I ratio is associated with obesity, metabolic syndrome, insulin resistance, and NAFLD[118]–[120]. ApoA-I is primarily produced by the liver in lipid poor forms. Phospholipids and cholesterols are added extracellularly through ATP binding cassette transporter A1 (ABCA1), a transmembrane protein present in both hepatocytes and peripheral tissues. The liver has been recognized as an essential site for initial lipidation involving a unique process called retroendocytosis[121]–[123]. This hepatic production of HDL could attenuate VLDL secretion via a phosphatidylinosital-3 kinase mediated signaling pathway[124]. Its effect in hepatic steatosis is yet to be determined.

Apolipoprotein A-V is a new member in the apolipoprotein family and has a specific impact on lipid metabolism[125]. ApoA-V is expressed in the liver, with a small amount secreted in VLDL and HDL[126]. In mouse models, apoa5-null mice displayed elevated serum TAG levels, while mice that overexpressed *apoA-V* had decreased serum TAG levels[125],[127]. These data suggest a role of apoA-V that is antagonistic to apoC-III in VLDL production, although mechanisms responsible for the apoA-V action remain to be defined. It was shown that the improvement in hepatic steatosis among obese NAFLD patients who underwent bariatric surgery was accompanied with significant reduction in hepatic *apoA-V* mRNA levels[128]. In HepG2 cells, knock down of apoA-V resulted in marked decrease in hepatic TAG content[128]. In cultured cells, apoA-V was found to be not in direct association with apoB100, but with intracellular lipid droplets[129]. In humans, two apoA-V polymorphisms have strong association with elevated serum TAG levels[130],[131]. However, it is unclear whether these polymorphisms offer protection against NAFLD.

Lipoprotein lipase (LPL) is an extracellular matrix bound enzyme present outside of capillary endothelial cells. Although not a member of apolipoproteins, it dictates the catabolism of chylomicron and VLDL, an integral process in normal lipoprotein metabolism[132]. LPL is produced by many tissues, including adipose tissue, cardiac and skeletal muscle, pancreatic islets, and macrophages, but not by the adult liver. LPL catalyzes the rate limiting step in the breakdown of TAGs from serum lipoproteins for utilization by the peripheral tissue (***Fig. 4***). An important activator of this enzyme is apoC-II, an exchangeable apolipoprotein produced by the liver. The genetic deficiency or malfunction of lipoprotein lipase results in type I hyperlipoproteinemia and subsequent ectopic TAG accumulation. In normal individuals, LPL activity in the adipose tissue is essential in buffering the circulatory TAG load, which protects against ectopic TAG accumulation, including hepatic steatosis[133]. In obesity, the impaired ability to up-regulate LPL by insulin exacerbates hepatic postprandial lipid load, thus causes hepatic steatosis. The regulation of LPL is therefore a crucial component in hepatic lipid homeostasis. Updates on the biology of LPL have been reviewed elsewhere[134],[135].

The expression of genes involved in hepatic lipid and lipoprotein metabolism is, to a large extent, regulated by an array of nuclear hormone receptors (also known as transcription factors) in response to changes in the balances of nutrients, mainly fatty acids and carbohydrates[136],[137]. Not surprisingly, expression of most of the above discussed apolipoproteins, LPL, and lipoprotein receptors is regulated by the nuclear hormone receptors, such as LXR[138], FXR[139], PPARα and PPARγ[140]. These nuclear hormone receptors are implicated in hepatic steatosis and some have been proposed as targets for treatment of NAFLD. LXR promotes free fatty acid uptake by the liver and promotes large VLDL secretion[141]. The *LXR*-null mice develop hepatomegaly with intrahepatic accumulation of cholesterol esters[142]. FXR, on the other hand is a bile acid sensor and decreases circulating TAGs upon activation[136],[137]. LXR and FXR are recognized as promising drug targets for an array of diseases including NAFLD. PPARα provides transcriptional control of genes in the β oxidation of fatty acids, whereas PPARγ is the principal regulator in adipogensis and promotes insulin sensitivity[143]. Fibrates are PPARα agonists that have been well established in treating dyslipidemia[144]. In rodent models, fibrates provide hepatic protection against diet induced NASH[145]. Thiazolidinediones, PPARγ agonists developed in the mid 1990s, were once among the frontline agents in treating type 2 diabetes mellitus[146]. In randomized control trials for NAFLD, pioglitazone was found to improve the liver enzyme profiles and histological grades of steatohepatitis, although there were no benefits in reducing fibrosis[147],[148]. These transcription factors have been reviewed[136],[137],[143],[149].

## CONCLUSION

Lipoprotein metabolism is a central process implicated in the development of hepatic steatosis. Our knowledge in the biology of lipoprotein metabolism has expanded significantly in the past two decades. Now key players in the system have been identified. We may begin to understand the molecular mechanism that modulates the formation, secretion, clearance and regulation of lipoprotein metabolism. Such knowledge will prepare us to better understand NAFLD, a growing problem that is endemic in the modern society.

Several key questions remain to be answered. Is lipoprotein metabolism primarily altered in NAFLD? If so, is that alteration pathogenic or is it simply a maladaptation secondary to metabolic syndrome? How does lipoprotein metabolism evolve as the progression of NAFLD? Are there changes in lipoprotein metabolism that one can identify in the serum that can predict disease progression? Finally, are there steps in lipoprotein metabolism that are amenable to intervention, thus halting or changing the course of disease? Issues raised by these questions will be addressed both on the basic science and clinical level and will surely impact the diagnosis and therapeutics in the future.
